# Variations in energy metabolism along the pericardium meridian and its relationship with visceral function adjustments during electroacupuncture

**DOI:** 10.1186/1472-6882-14-323

**Published:** 2014-08-30

**Authors:** Shu-Xia Zheng, Xiao-Hua Pan, Jin-Sen Xu, Chun-Ying Xiu, Ya-Qin Dong, Xiaoxiang Zhu

**Affiliations:** Fujian Academy of Traditional Chinese Medicine, Class III Laboratory of Acupuncture Physiology, Key Unit of the Propagated Sensation along Meridian of State Administration of Traditional Chinese Medicine, Fuzhou, Fujian 350003 China

**Keywords:** Pericardium meridian, Microcirculatory blood perfusion, Transcutaneous oxygen partial pressure, Visceral function, Meridian

## Abstract

**Background:**

Electroacupuncture (EA) is a traditional Chinese medicine treatment guided by meridian theory. As it gradually gains more worldwide acceptance, a clarification of its mechanisms is extremely urgent. We observed variations in transcutaneous oxygen pressure/carbon dioxide pressure (_*tcp*_O_2_/_*tcp*_CO_2_) and microcirculation blood perfusion units (BPU) along the pericardium meridian, and cardiac function during EA at Neiguan (PC6) to explore variations in energy metabolism and its relationship with visceral function adjustments during EA.

**Methods:**

Twenty-two healthy volunteers participated in this study. Three channel laser Doppler flowmetry and _*tcp*_O_2_/_*tcp*_CO_2_ detection systems were used to detect _*tcp*_O_2_/_*tcp*_CO_2_ and microcirculation BPU along the pericardium meridian. A hemodynamic monitor was used to detect cardiac function.

**Results:**

In the normal state, the microcirculatory BPU along the pericardium meridian were significantly higher than that of their bilateral corresponding control points (p < 0.05). During EA at PC6, the values of the microcirculatory BPU along the pericardium meridian did not vary, and few increased. In the normal state, the values of _*tcp*_O_2_ along the pericardium meridian were significantly higher than those of their bilateral corresponding control points (p < 0.05). In addition, the values of _*tcp*_CO_2_ along the pericardium meridian were lower than those of their bilateral corresponding control points. In comparison with the normal state, EA could decrease _*tcp*_O_2_ along the meridian significantly (p < 0.05) and increase _*tcp*_CO_2_. During EA at PC6 in healthy volunteers treated by artificial acute mild hypoxia, cardiac output and cardiac index (p < 0.05) decreased and systemic vascular resistance increased significantly (p < 0.05).

**Conclusions:**

In the normal state, the values of microcirculatory BPU and _*tcp*_O_2_ along the pericardium meridian were both higher than those of their bilateral corresponding control points. Energy metabolism was vigorous along the meridian. During EA, the decrease in oxygen partial pressure along the pericardium meridian might be a result of strengthened energy metabolism of associated tissue and increased oxygen consumption. The variations in energy metabolism along the pericardium meridian during the course of EA had a close relationship with visceral function adjustments.

**Trial registration:**

Chinese Clinical Trial Registry ChiCTRTRC13003193.

## Background

Holism is an important concept in traditional Chinese medicine (TCM), which holds that the human body is an organic whole and that the body surface and internal organs are interrelated and mutual. Lesions of internal organs can manifest as external symptoms, so we can deduce changes in the internal organs by observing the external body. The meridian is an important pathway of contact and communication between the inside and outside of the human body. Inside the body, the meridian links with viscera, while outside of the body it connects the limb joints. The meridian promotes Qi and blood, nourishes Yin and Yang, and responds to reactions outside of the body. Acupuncture stimulates acupoints, which communicate along the meridians until arrival at the organs (diseases or effective organs). There are then a series of changes, not only in the meridian, but also in the corresponding organs’ function during the course of acupuncture. More than 2000 years ago, ancient Chinese physicians noted the existence of a relationship between the meridians and organs; the regulation of the acupoints’ effects has been summarized by clinical practice. However, because of the different systems in Eastern and Western medicine, it is difficult to properly interpret the mechanism of acupuncture under the guidance of meridian theory by Western medicine, which is built on the research methods of anatomy and analysis. Although acupuncture treatment has been accepted in more than 100 countries, relationships among acupoints, meridians, and corresponding organs have not been fully clarified. Therefore, changes in meridians during acupuncture, changes in the corresponding organ functions, and whether the two changes are related or synchronized are the focus of this study. We studied the changes in energy metabolism of the meridians and the corresponding changes in organ function during EA. We hope to provide an experimental basis to further explore the relationship between the meridians and viscera and the mechanism of EA.

## Methods

### Volunteers

Twenty-two healthy volunteers (fifteen male, seven female), aged 22 ± 3 years, height 156–174 cm, body mass 45–63 kg, were college students recruited from Fujian University of Traditional Chinese Medicine. No volunteers had a history of chronic disease and all gave informed consent. The study was approved by Chinese Medicine Clinical Research Ethics Committee of Fujian Academy of Traditional Chinese Medicine. All detections were finished in the Class III Laboratory of Acupuncture Physiology, Fujian Meridian Institute of Traditional Chinese Medicine, Key Unit of the Propagated Sensation along Meridian of State Administration of Traditional Chinese Medicine.

### Detection instruments

The three-channel laser Doppler flowmetry and _*tcp*_O_2_/_*tcp*_CO_2_ detection system (PeriFlux System 5000, Perimed, Järfälla, Sweden) had a wavelength of 780 nm and time constant 0.2. The probe model used to detect the surface microcirculation BPU was 412, while the probe used to detect deep microcirculation BPU was 418–1. The detection system has three channels that can detect the surface or deep microcirculation BPU and _*tcp*_O_2_/_*tcp*_CO_2_ of the meridian and its bilateral control points, contemporarily and continuously in the same day. The data are stored on a computer. Hemodynamic monitor (model BZ-4110-121, CardioDynamics, San Diego, CA, USA) was used to record 16 cardiac function indexes such as CO, CI, and SVR.

### Detection parts

**Figure 1:**
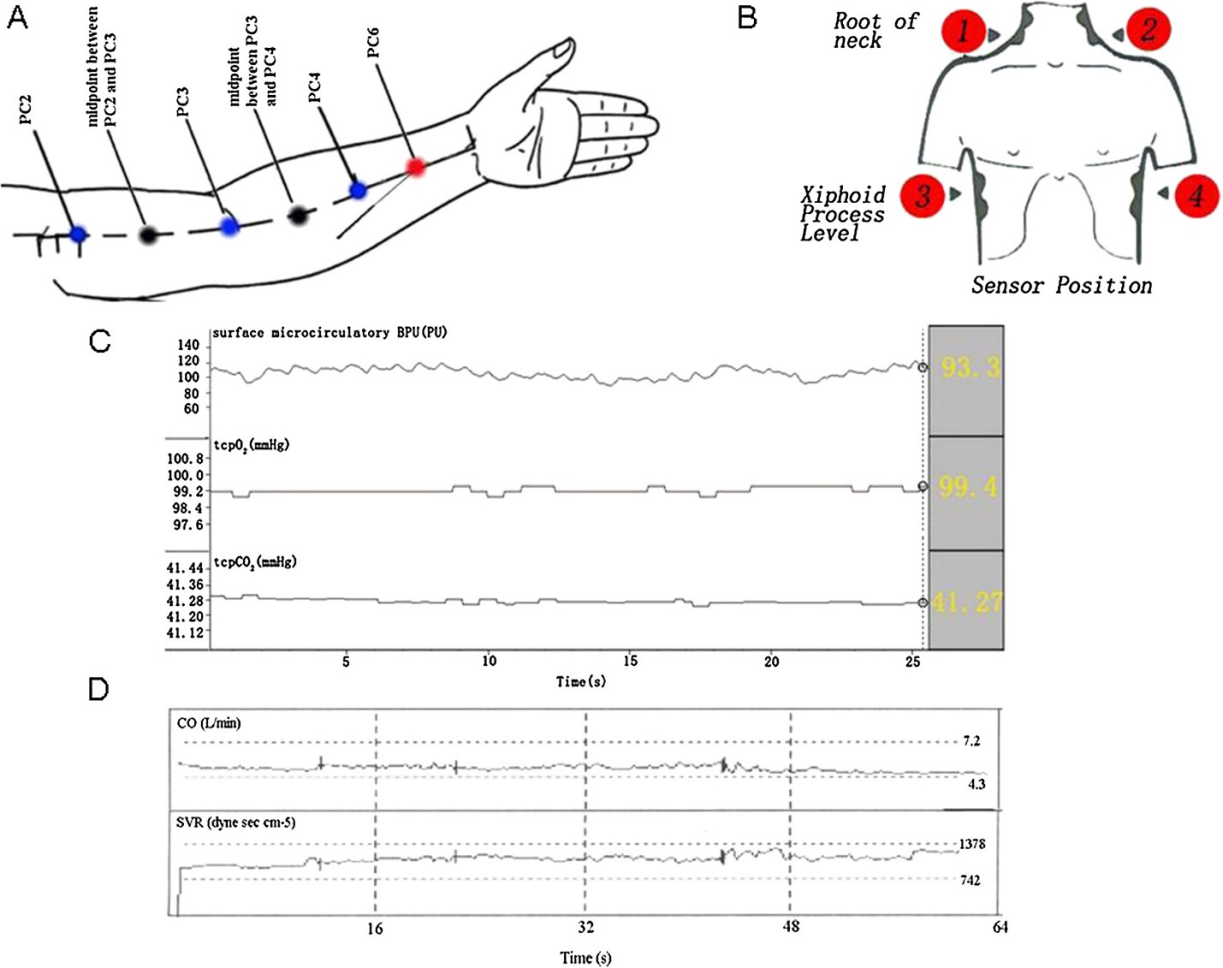
**Illustration of study design. (A)** Detection points of microcirculation BPU and _*tcp*_O_2_/_*tcp*_CO_2_. PC2, Tianquan acupoint, PC3, Quze acupoint, PC4, Ximen acupoint, PC6, Neiguan, and EA point. **(B)** Sensor position of the cardiac function detection. **(C)** Original signal of microcirculation BPU and _*tcp*_O_2_/_*tcp*_CO_2_. **(D)** Original signal of hemodynamic monitor.

The detection parts were Ximen (PC4), Quze (PC3), Tianquan (PC2), the midpoint between PC4 and PC3 (non-acupoint), and the midpoint between PC3 and PC2 (non-acupoint) of the pericardium meridian. The EA point was Neiguan (PC6). All the detection parts are shown in Figure 1A.

### Detection procedure

Each volunteer was studied once every 3–4 days at an ambient room temperature of 28 ± 1°C [1]. All detection instruments were turned on to preheat for 20 minutes and to keep the instruments steady. Instruments were checked while the volunteers adapted to the laboratory. When the microcirculation BPU and _*tcp*_O_2_/_*tcp*_CO_2_ were detected, the volunteers lay on their backs, the detected position was exposed, and the skin was regularly disinfected and degreased with 75% alcohol. The probes to detect the _*tcp*_O_2_/_*tcp*_CO_2_ and microcirculation BPU were placed at the detected positions separately. During the next observation, the positions of the probes for _*tcp*_O_2_/_*tcp*_CO_2_ and microcirculation BPU were switched. When cardiac functions were detected, sensors were fixed in the root of the neck and xiphoid process (Figure 1B). Sixteen cardiac function indexes, such as cardiac output (CO), cardiac index (CI), and systemic vascular resistance (SVR), were recorded as control by a hemodynamic monitor. Then, the volunteer inhaled low oxygen mixed gas (14% O_2_ and 86% N_2_) for 30 minutes to imitate acute mild hypoxia similar to 3000 m above sea level. EA was conducted when the mixed gas was inhaled for 10 minutes. The changes in each index before EA, during EA, and after EA were recorded separately. EA was conducted at PC6 with a frequency of 0.5 Hz, a wave width of 0.2 ms, and an intensity of 3–4 V. EA was sustained for 10 minutes. The average values during the 10 minutes were taken as the values during EA. The whole detection process is illustrated in Figure 1.

### Statistical analysis

Data are represented as the mean ± SD and analyzed by SPSS 12.0 (SPSS, Chicago, IL, USA). To avoid the deviation from the weather, the data of cardiac function was represented with a ratio, which comes from the detection value compared with the control value. Groups were compared with single factor analysis of variance, compared with the value before and after EA by using a paired *t*-test; p-values < 0.05 were considered as statistically significant.

## Results

### Comparison of surface microcirculation BPU values of the pericardium meridian and its bilateral control points in the normal state

**Figure 2:**
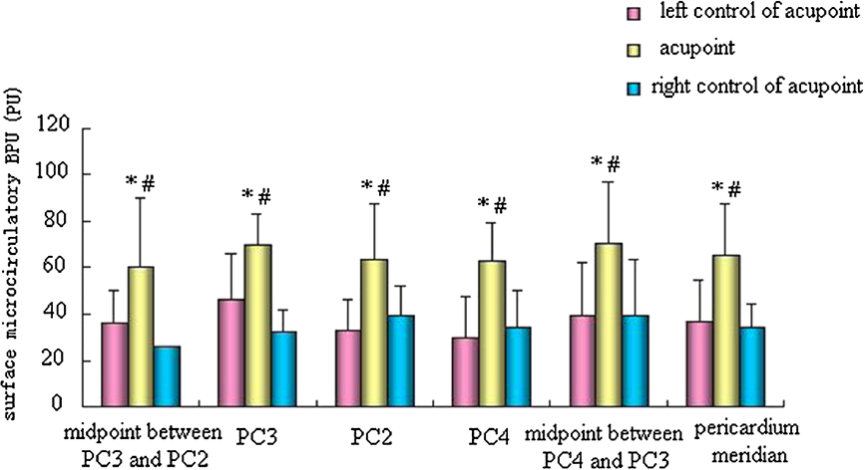
**Comparison of surface microcirculation BPU of the pericardium meridian and its bilateral control points in the normal state.** *p < 0.05 vs. left control of acupoint groups; # p < 0.05 vs. right control of acupoint groups.

We detected PC4, PC3, PC2, the midpoint between PC4 and PC3 (non-acupoint), and the midpoint between PC3 and PC2 (non-acupoint) of the pericardium meridian and its bilateral control points. We found that the surface microcirculation BPU values of the three acupoints and two non-acupoints of the pericardium meridian were significantly higher than those of the bilateral control points (p < 0.05) (Figure 2).

### Comparison of _*tcp*_O_2_/_*tcp*_CO_2_of the pericardium meridian and its bilateral control points in the normal state

**Figure 3:**
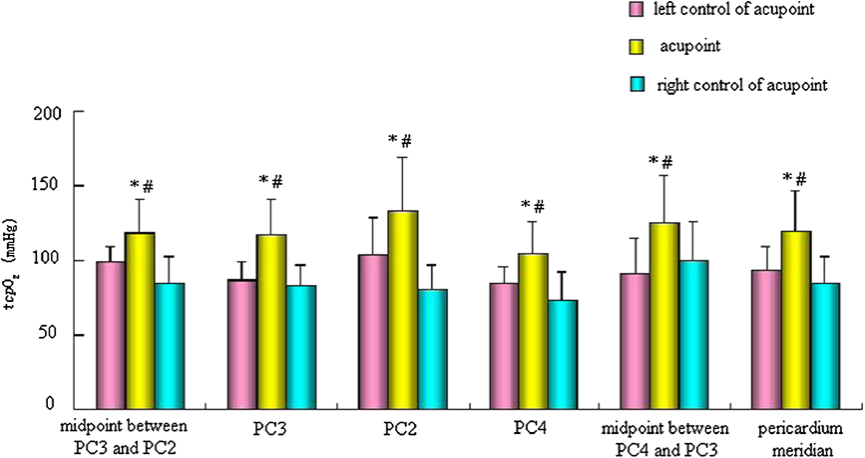
**Comparison of**_***tcp***_ **O**_**2**_ **of the pericardium meridian and its bilateral control points in the normal state.** *p < 0.05 vs. left control of acupoint groups; # p < 0.05 vs. right control of acupoint groups.

**Figure 4:**
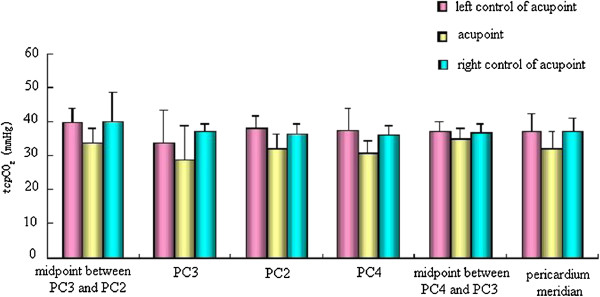
**Comparison of**_***tcp***_ **CO**_**2**_ **of the pericardium meridian and its bilateral control points in the normal state.**

We detected _*tcp*_O_2_/_*tcp*_CO_2_ of PC4, PC3, PC2, the midpoint between PC4 and PC3 (non-acupoint), and the midpoint between PC3 and PC2 (non-acupoint) of the pericardium meridian and its bilateral control points, and found that _*tcp*_O_2_ of three acupoints and two non-acupoints of the pericardium meridian were significantly higher than those of the bilateral control points (p < 0.05), while the _*tcp*_CO_2_ of three acupoints and two non-acupoints of the pericardium meridian were lower than those of the bilateral control points (Figures 3 and 4).

### Effect of EA on the surface microcirculation BPU values of the pericardium meridian and its bilateral control points

**Figure 5:**
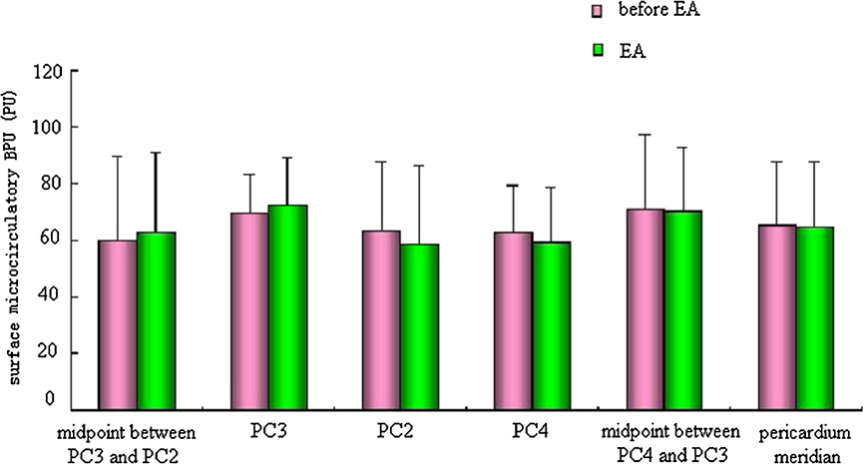
**Effect of EA on the surface microcirculation BPU values of the pericardium meridian and its bilateral control points.**

The effect of EA at PC6 on the surface microcirculation BPU values was not significant. Some were a little higher than that before EA, but the difference was not statistically significant (Figure 5).

### Effect of EA on _*tcp*_O_2_/_*tcp*_CO_2_of the pericardium meridian and its bilateral control points

**Figure 6:**
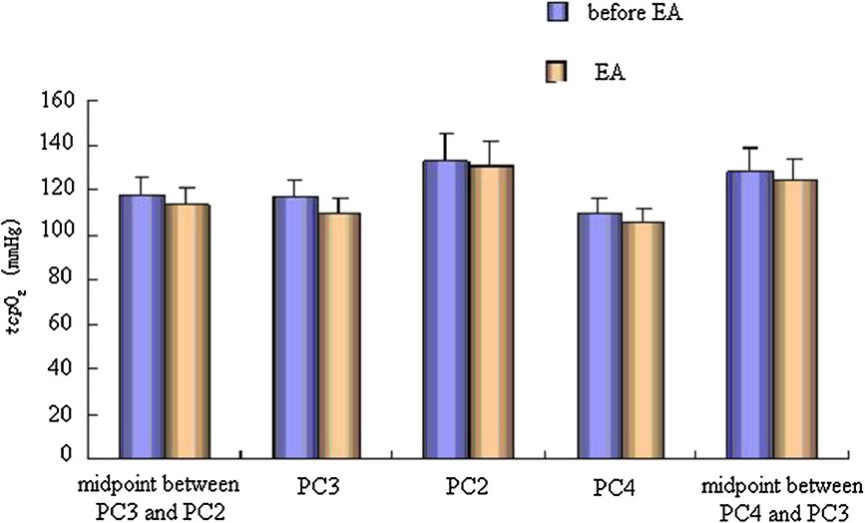
**Effect of EA on**_***tcp***_ **O**_**2**_ **of the pericardium meridian and its bilateral control points.**

**Figure 7:**
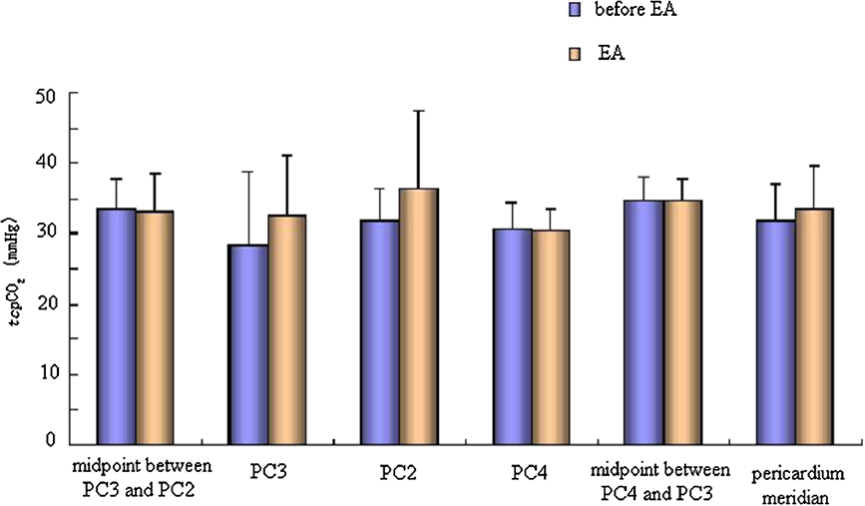
**Effect of EA on**_***tcp***_ **CO**_**2**_ **of the pericardium meridian and its bilateral control points.**

The effect of EA at PC6 on _*tcp*_O_2_ was not same for the four acupoints and the two non-acupoints. Compared with that before EA, EA could decrease _*tcp*_O_2_ with no significant difference, while the _*tcp*_O_2_ of the bilateral control points had no obvious changes before and after EA. Meanwhile EA could increase _*tcp*_CO_2_ with no significant difference (Figures 6 and 7).

### Effect of EA at Neiguan (PC6) on cardiac functions

**Figure 8:**
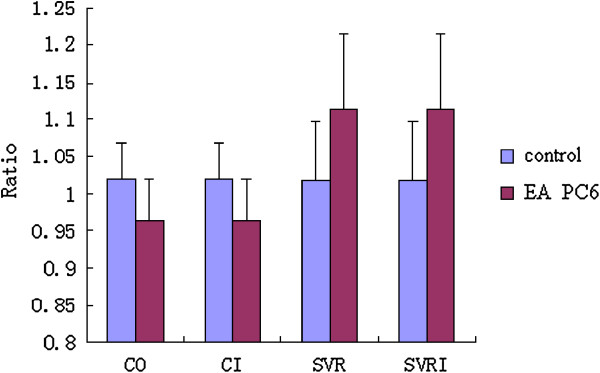
**Effect of EA at PC6 on cardiac functions.** The results are showed as a ratio. The ratio was calculated by comparing the detection value to the control value.

CO and CI increased after oxygen inhalation, while EA at PC6 decreased CO and CI significantly. SVR and systemic vascular resistance index (SVRI) decreased after oxygen inhalation, while SVR and SVRI increased significantly (p < 0.05) during EA at PC6 (Figure 8).

## Discussion

Meridian theory is a core theory of TCM. It guides clinical practice and embodies all aspects of TCM, such as physiology, pathology, diagnosis, and treatment. As *Lingshu Jingmai* said, the twelve meridians were the reason why human beings are born, why diseases occur, why humans get well, and where the new learner starts and the work stops. Therefore, as meridian theory is the guiding principle of TCM basic theories, a clarification of the meridians will further develop TCM and medical science. Some studies have focused on the optical, biochemical, or nerve electrophysiology characteristics of the meridians [2–4]. All studies are trying to discover a mechanism of meridians from a different point of view.

Our previous studies showed that, in the normal state, the infrared radiant track along meridian (IRRTM) is basically consistent with classical meridians that can be observed on the human body surface. This indicates that there must be a corresponding material basis for meridians [5]. We also found that the energy metabolism on the meridian was higher than that on the non-meridian control points and could be strengthened by EA, and the meridian takes part in the corresponding visceral function adjustments as a whole [6–11]. However, it has been difficult to properly explain all the above facts by the known mechanism of neurohumor comprehensive regulation, which indicates that there are other unknown ways to make adjustments to the human body.

Energy metabolism is the most basic character of life and is required to maintain all life activities. Energy metabolism also varies for different structures and tissues. The meridians act as contacts, adjustments, and response systems within these tissues, which includes energy metabolism. Microcirculation is the blood circulation between arterioles and venules, and its basic function is to exchange substances in blood and tissue fluid. There is a close association between microcirculation and the meridians. Some studies showed that the Qi that runs in the meridians is closely related with _*tcp*_O_2_/_*tcp*_CO_2_, which is a measure of energy metabolism [12, 13]. Therefore, we used _*tcp*_O_2_/_*tcp*_CO_2_ and microcirculation BPU to further analyze the energy metabolism of meridian tissue. We found that the values of microcirculation BPU and _*tcp*_O_2_ of the meridians were higher than those in the bilateral control points, which indicated that more blood ran through the meridians and the oxygen content was higher. Therefore, the energy metabolism was higher, which could make it possible for meridians make functional adjustments in the human body [14–17].

The effects of EA are more obvious in pathological or dysfunctional conditions than in physiological conditions. This is consistent with the theory that EA has the ability to “rectify deviations” [18]. Therefore, we had volunteers inhale low oxygen mixed gas (14% O_2_ and 86% N_2_) to imitate acute mild hypoxia from about 3000 m elevation above sea level. As a result, the condition of the volunteers deviated from normal, and the effect of EA was detected more clearly. We found that CO and CI increased after oxygen inhalation, while EA at PC6 could decrease CO and CI significantly. SVR and SVRI decreased after oxygen inhalation and increased significantly (p < 0.05) during EA at PC6, which reflects the adjustments of EA acupoints of the pericardium meridian on cardiac functions.

We also found that, in the normal state, the values of _*tcp*_O_2_/_*tcp*_CO_2_ and microcirculation BPU along the pericardium meridian were stable. When acupoints were stimulated by EA, the values of the skin microcirculation BPU remained steady or increased slightly, while the values of _*tcp*_O_2_ decreased. This change indicated that decreases in _*tcp*_O_2_ values were not from decreases in microcirculation BPU values, but from increases in energy metabolism and oxygen consumption caused by EA [19]. We also confirmed that EA took effect through increased use of oxygen and energy metabolism. The microcirculation BPU values of the pericardium meridian were higher than those of the non-meridian, which could be increased slightly by EA. Therefore, energy metabolism in the meridian was stronger, which created a good condition for the meridian to take part in the functional adjustment of the human body. We hypothesized that EA could not only change the meridian energy metabolism but also adjust the corresponding visceral function. As a connection, adjustment, and response system, the meridians have a special structure. However, we have not found the material basis. Meridians may be channels of multiple structures that transform and transmit materials, energy, and messages.

## Conclusions

Our study showed that the microcirculatory BPU values and _*tcp*_O_2_ along the pericardium meridian were both higher than those of their bilateral corresponding control points, which indicated that energy metabolism was vigorous along the meridian in the normal state. During EA, decreased oxygen pressure along the pericardium meridian might be a result of strengthened energy metabolism of associated tissue and increased oxygen consumption. Variations in energy metabolism along the pericardium meridian during EA are closely related with visceral function adjustments.

## Abbreviations

_*tcp*_O_2_/_*tcp*_CO_2_: transcutaneous oxygen pressure/carbon dioxide pressure
BPU: Blood flow unit
PSM: Propagated sensation along the meridian
CO: Cardiac output
CI: Cardiac index
SVR: Systemic vascular resistance
SVRI: Systemic vascular resistance index
EA: Electroacupuncture
IRRTM: Infrared radiant track along meridian.
